# Predictive Psychosocial Factors of Child-to-Parent Violence in a Sample of Mexican Adolescents

**DOI:** 10.3389/fpsyg.2020.576178

**Published:** 2020-11-16

**Authors:** Cristian Suárez-Relinque, Gonzalo del Moral Arroyo, Teresa I. Jiménez, Juan Evaristo Calleja, Juan Carlos Sánchez

**Affiliations:** ^1^Department of Education and Social Psychology, Pablo de Olavide University, Seville, Spain; ^2^Department of Psychology and Sociology, University of Zaragoza, Teruel, Spain; ^3^Faculty of Psychology, Autonomous University of Nuevo León, San Nicolás de los Garza, Mexico

**Keywords:** problematic use of social networking sites, family communication, psychological distress, perceived non-conformist social reputation, child-to-parent violence

## Abstract

The aim of this study was to carry out a psychosocial analysis of child-to-parent violence (CPV) in a sample of school adolescents, considering a set of individual variables (psychological distress, problematic use of social networking sites, and perceived non-conformist social reputation) and family variables (open and problematic communication with parents) according to sex. The sample consisted of 3,731 adolescents (54% boys), aged between 14 and 16 years (M = 14.6 years, SD = 0.567), from the state of Nuevo León, Mexico. The scores of the boys and girls were analyzed to check for differences. Also, correlations between all the study variables were calculated. Finally, a multiple stepwise regression analysis was carried out for the total sample and also for boys and girls separately. Results confirmed the important role of individual variables as predictors of CPV in boys and girls. The main difference between boys and girls was observed in the predictive weight of problematic use of social networking sites, which was higher in girls than in boys. Open communication with the father was a significant factor for predicting the decrease of CPV levels in the case of boys, while open communication with the mother predicted the decrease of CPV in girls. Problematic communication with the mother showed similar values in boys and girls when predicting CPV, however, the predictive weight of problematic communication with the father was higher in girls than in boys. These results are interesting and have important implications for the prevention of CPV.

## Introduction

Child-to-parent violence (hereinafter “CPV”) is defined as any repeated harmful act (physical, psychological, or economic) carried out by children against their parents or any other figure occupying their role of authority, with the main and ultimate objective of gaining power and/or control over them, also achieving different specific objectives (material or otherwise) during the process (Llamazares et al., 2013; Holt, 2016).

In terms of the prevalence of this problem in adolescents, data available in scientific literature are extremely disparate due to the different definition and measurement criteria used when analyzing this problem (Holt, 2016). The rates registered in different countries show percentages between 45 and 95% in the case of verbal violence and between 4.6 and 22% in the case of physical assault perpetrated at least once a year (Condry and Miles, 2014; Lyons et al., 2015; Calvete and Orue, 2016; Suárez-Relinque et al., 2019). Regarding economic CPV, few studies have reported data on this type of violence but the available information indicates percentages of prevalence ranging between 29 and 60% for damage to property and at 15.8% in the case of stealing (Condry and Miles, 2014; Margolin and Baucom, 2014; Rico et al., 2017; Arias-Rivera and Hidalgo, 2020; Contreras et al., 2020). Considering data by country, prevalence of physical CPV (PCPV) in the United States and Canada ranges between 11 and 22%, while verbal CPV (VCPV) ranges between 51 and 75% (Pagani et al., 2009; Margolin and Baucom, 2014). In Spain, the prevalence of PCPV is approximately 8%, while for VCPV the prevalence rate is around 90% (Calvete et al., 2015a,b). In the specific case of Mexican adolescents, prevalences of around 80% have been observed for verbal violence and 7% for physical violence (Calvete and Veytia, 2018; Cancino-Padilla et al., 2020). Finally, regarding the age range of the aggressor, this may be established at between 4 and 24 years, although most cases occur in middle adolescence (14–17 years), progressively decreasing as age increases (Ibabe and Bentler, 2016; Simmons et al., 2018).

Regarding the main predictive factors of CPV, scientific literature has identified different dimensions at individual level that reveal a direct relationship with CPV. In this sense, depressive symptoms have been regularly described (Castañeda et al., 2012; Calvete et al., 2013a; Ibabe et al., 2014), as well as problems related to the consumption of alcohol and other drugs (Calvete et al., 2011, 2015b; Ibabe and Jaureguizar, 2011), alexithymia (Martínez-Ferrer et al., 2018a), a low level of empathy (Ibabe and Jaureguizar, 2011), narcissism (Calvete et al., 2015b), and low self-esteem (Ibabe and Jaureguizar, 2011; Loinaz et al., 2017). Interestingly, prior research has identified another set of individual dimensions that have also been shown to be important in the field of violence between peers, but which, having received little attention in the specific field of CPV, do not allow conclusions to be drawn regarding their role in this problem. Examples include psychological distress (PD), problematic use of social networking sites (PUSNSs), and perceived non-conformist social reputation (PNCSR), dimensions that have been analyzed mainly in the field of violence between peers. In relation to this, it is also important to highlight the link that some researchers point between peer violence and CPV. For example, a recent study by Carrascosa et al. (2018) compared violent behaviors toward peers in adolescents committing CPV and adolescents without CPV problems. The results of this study showed that the minor offenders committing CPV exert more violence toward their peers than adolescents without problems of CPV. Considering this observed relationship between both types of violence, it may be worthwhile to investigate whether the dimensions with demonstrated importance in violence between peers play a similar role in CPV.

PD is defined as psychological suffering expressed through symptoms of anxiety and depression, from mild to severe, with a variable degree of deterioration in the behavioral, cognitive, or emotional dimensions of functioning (Castro et al., 2019). Few studies have used this dimension in the field of violence in adolescence, although some interesting research can be found on violence between peers, albeit reporting contradictory results. Specifically, some studies describe that aggressors display higher levels of PD (anxiety and stress) than levels commonly found in adolescents (Carlson and Corcoran, 2001; Sánchez-Sosa et al., 2010; Romero et al., 2019), while others report no significant differences in aggressors with respect to ordinary adolescents (Brendgen et al., 2004; Estévez et al., 2005). As regards the relationship between PD and CPV, few studies have addressed this issue. For example, Kennedy et al. (2010) found that adolescents who were violent toward their parents had experienced greater PD than those who had not suffered from that problem. Also, Lozano et al. (2013) analyzed the link between CPV and PD, finding a positive correlation between both variables. Calvete et al. (2014a) explored the characteristics of CPV in Spain based on the speech of parent-abuse offenders, their parents, and the professionals in this area. The results of their study pointed to emotional stress in children as a relevant predictor of CPV.

The PUSNSs can be defined as the prolonged and compulsive use of social networks that undermines other social activities, studies, work, interpersonal relationships, and the psychological health and well-being of the subject (Andreassen and Pallesen, 2014). This problem generally affects populations that are vulnerable due to their age, such as adolescents (Pallanti et al., 2006; Puerta-Cortés and Carbonell, 2014). Recent research includes many studies that have analyzed the relationship between the aforementioned variable and violence between peers (Martínez-Ferrer and Moreno-Ruiz, 2017; Martínez-Ferrer et al., 2018b), cyberbullying (Giménez et al., 2015), and cybervictimization (Blanco, 2014; Martín et al., 2016). However, as far as the literature reviewed is concerned, very little information is available about the association between PUSNS and CPV. One of the few studies to provide data in this regard is the one conducted by Martínez-Ferrer et al. (2018a), who described a positive correlation between both variables, observing that high levels of CPV corresponded to high PUSNS levels.

In the case of PNCSR, as with the variables mentioned above, their study has been limited almost exclusively to the field of school violence (Buelga et al., 2012; Estévez et al., 2014). PNCSR can be more specifically defined as the adolescent’s perception of his or her own social image as an image based on a continual transgression of established social rules and a defiance of formal institutions (Estévez et al., 2008; Moreno et al., 2012). This dimension is positively related to adolescents’ perception of their social reputation. In other words, the more challenging, harsh, and rebellious adolescents perceive themselves, the more favorable their perception of their own social reputation will be. In this sense, non-conformist self-perception is a risk factor for the adolescent’s participation in violent behavior, which is understood as a form of transgression that allows the individual to achieve social recognition (Estévez et al., 2014; Buelga et al., 2015; Romero et al., 2019). So far, few studies in the field of CPV have included this variable in their analysis, but the available results point in the same direction as those observed in the school context, indicating a positive relationship between CPV and PNCSR. For example, Del Moral et al. (2019) found that adolescents who display the highest levels of PNCSR are, in turn, the ones who present the highest levels of violence against their parents. Also, in the study developed by Terceño (2017), adolescents from families with high levels of CPV scored higher in PNCSR than those who came from families with medium and low level of CPV.

On the other hand, in terms of family environment, different risk factors related to the onset of CPV in adolescence have also been identified in previous research. For example, lack of emotional support from parents (Ibabe et al., 2013; Calvete et al., 2014b, 2015b; Suárez-Relinque et al., 2019), low family cohesion, or high levels of conflict (Jaureguizar et al., 2013; Ibabe and Bentler, 2016; Zuñeda et al., 2016). Likewise, recent research has reported that parental socialization styles in which the lack of emotional support from parents and problems in communication with children coexist, facilitate the onset of CPV during adolescence (Calvete et al., 2013a, 2015b; Ibabe and Bentler, 2016; Simmons et al., 2018). This is the case with so-called authoritarian and neglectful parental styles. In contrast, parental styles characterized by open communication between parents and children and high levels of emotional support (indulgent and authoritative styles) have been identified as the most protective against CPV (Beckmann et al., 2017; Garaigordobil, 2017; García et al., 2018; Suárez-Relinque et al., 2019).

In short, different studies in recent years have analyzed and verified the importance of emotional support and positive communication between parents and children as protective factors against CPV in adolescence. Nevertheless, one clarification should be made regarding the information available in the above-mentioned research with respect to family communication (FC) and its relationship with CPV. Firstly, previous CPV studies have analyzed FC mostly as an aspect integrated in the study of parental socialization practices (Calvete et al., 2015b; Beckmann et al., 2017; García et al., 2018). In this sense, FC has been explored and defined as the more or less habitual use that parents make of dialog and reasoning when transmitting their decisions to their children. Thus, the reviewed studies highlighted the preventive value of those styles in which emotional support is used together with dialog and reasoning when transmitting parental practices. However, there is a lack of information regarding the specific role played by the dimensions of FC (problematic communication and open communication) in the development of CPV in adolescence, and only few studies have addressed this goal. For example, in the study by Contreras and Cano-Lozano (2014a), it is observed that parent-abuse offenders reported having less openness and higher levels of problematic communication with parents (especially with the mother) than the other delinquent and normal adolescents. Considering the information stated here, it would be worthwhile to deepen knowledge of the relationship between these dimensions of FC and CPV.

Finally, in relation to socio-demographic factors, attention should be drawn to the importance of considering the sex variable in the study of CPV in adolescence, taking into account the differences observed in the results of previous research. In this sense, it is also important to point the disparity found in the results, mainly depending on the sample used. For example, in studies with community samples, similar rates of global CPV have been observed for boys and girls, and even higher levels in girls (Jaureguizar et al., 2013; Ibabe, 2015; Calvete and Veytia, 2018). Some studies indicate that verbal aggression is more frequent in girls, while physical aggression is more used by boys (Pagani et al., 2009; Calvete et al., 2013b; Jaureguizar et al., 2013; Calvete and Orue, 2016; Beckmann et al., 2017). However, other studies that also used community samples found no significant differences between boys and girls on the type of CPV exerted (Elliott et al., 2011; Calvete et al., 2015b; Ibabe and Bentler, 2016). In the case of judicial and clinical samples, most studies have reported higher rates of aggression in boys than in girls (Boxer et al., 2009; Walsh and Krienert, 2009; Routt and Anderson, 2011; Condry and Miles, 2014; Contreras and Cano-Lozano, 2014b; Ibabe et al., 2014; Gallego et al., 2019; Loinaz et al., 2020). According to these studies, physical aggression is more used by boys (Boxer et al., 2009; Walsh and Krienert, 2009; Routt and Anderson, 2011), although there are no significant differences between boys and girls regarding the severity of the assault (Condry and Miles, 2014; Simmons et al., 2018; Loinaz et al., 2020).

As regards the gender differences in the rest of the variables, compared to boys, girls tend to show higher of PD (Mewton et al., 2016; Van Droogenbroeck et al., 2018; Zhang et al., 2018) and PUSNS (Sarabia and Estévez, 2016; Martínez-Ferrer et al., 2018b; Aparicio et al., 2020). In contrast, higher levels have been observed in boys in the case of PNCSR (Buelga et al., 2012; Shin, 2017). In terms of FC, few studies have provided information on both the sex of the adolescent and the type of communication (open or problematic) with their parents. Furthermore, available findings present conflicting results. Even so, a review of recent literature seems to confirm the existence of significant differences according to sex. In general, boys show slightly higher levels than girls in open communication with their fathers (OCF) and girls slightly higher levels in open communication with their mothers (OCM) and problematic communication with the father (PCF) and mother (PCM; Parra and Oliva, 2002; Cava, 2003; Keijsers and Poulin, 2013).

### The Present Study

Taking into account the background information presented in the previous section, this study aimed to identify predictive variables of CPV in the individual (PUSNS, PD, and PNCSR) and family (FC), according to the sex of the adolescent. To accomplish this general goal, we address four specific objectives: first, to analyze the differences in the study variables between boys and girls: second, to explore the relationships between all the study variables; third, to estimate the relative importance of PUSNS, PD, PNCSR, and FC in the prediction of CPV; and fourth, to explore sex-based differences in the relative importance of PUSNS, PD, PNCSR, and FC in the prediction of CPV.

This study aimed to deepen knowledge of the individual and family factors that explain CPV. Regarding the main predictive factors of CPV, firstly, it should be reminded that the causes of behavioral problems in adolescence are multiple and can be found at individual and social level (Estévez et al., 2008). In previous research has been highlighted the importance of considering not only individual factors but also those linked to the social environment to which the adolescent belongs, in order to get a better understanding of violent behavior in adolescence (Estévez et al., 2008; Martínez-Ferrer et al., 2011; Jiménez and Estévez, 2017). In this sense, dimensions from the family context have been shown as specially relevant to address the study of CPV. In the present study, the role of FC dimensions is explored. It has to be pointed that, until now, FC has only been mostly analyzed in the field of CPV in its role as a transmitter of parental practices, integrated into parental socialization styles. On the other hand, the relevance of the dimensions of FC (problematic communication and open communication) has been shown in the field of violence between peers, therefore it could be interesting to analyze the importance of these dimensions in the field of CPV.

One of the most noteworthy contributions of the present study is the incorporation in the analysis of CPV of individual dimensions that have thus far been insufficiently examined by researchers. These dimensions include PUSNS, PD, and PNCSR. As with the familiar variables mentioned above, the previous research has routinely identified these variables as risk factors for violence between peers, but their importance for predicting CPV is barely known.

Finally, it is important to highlight that the choice of the variables and objectives of the present study were based not only on the gaps detected in literature but also on the link that, according to some researchers, exists between violence between peers and CPV (see Carrascosa et al., 2018). Considering this, it may be worthwhile investigating whether the dimensions with demonstrated importance in violence between peers play a similar role in CPV.

## Materials and Methods

### Participants

The study involved a total of 3,731 adolescents (54% boys), aged between 14 and 16 (M = 14.6 years, SD = 0.567), from the state of Nuevo León, Mexico. Adolescents were selected from 89 educational centers located in the Nuevo León region (Mexico). Selection was performed by means of stratified random sampling that considered the geographical area and the type of ownership. 60.44% of the participants came from urban schools and 87.7% studied at public educational centers (Table 1). Missing data were processed using the listwise deletion procedure.

**Table 1:** Sociodemographic variables.

Variables	Total sample	Sex	
		Boys	Girls
Age			
14	2,131 (57.1%)	1,142 (53.6%)	989 (46.4%)
15	986 (26.4%)	555 (56.3%)	431 (43.7%)
16	614 (16.4%)	316 (51.5%)	298 (48.5%)
Geographical area			
Urban	2,253 (60.4%)	1,240 (55%)	1,013 (45%)
Rural	1,478 (39.6%)	773 (52.3%)	705 (47.7%)
School ownership			
Public	3,272 (87.7%)	1746 (53.4%)	1,526 (46.6%)
Private	459 (12.3%)	267 (58.2%)	192 (41.8%)
Total	3,731 (100%)	2013 (54%)	1718 (46%)

### Procedure

The selection of the educational centers, as well as the planning and development of the field work, was carried out jointly by the Autonomous Universities of Nuevo León in Mexico and Pablo de Olavide in Seville. The research team contacted the management of the selected centers to formally request their participation in the study. Once the schools’ participation was confirmed, the researchers requested the voluntary collaboration of the students and the written consent of their families. The data were collected between March 2018 and May 2018. The questionnaire was administered by the researchers in the classrooms, where the adolescents usually received classes. The study took the respondents approximately 60 min to complete all the scales included in the questionnaire. During the administration of the questionnaire, the students were informed that their participation was anonymous and that they could abandon the session at any time without completing the questionnaire. Lastly, it is important to underline that this research was approved by the Ethics Committee of the Pablo de Olavide University in Seville and was carried out respecting the fundamental principles of the Declaration of Helsinki.

### Materials

Instruments used in the present study have been adapted into Spanish language using the parallel back-translation method (Brislin, 1986). Also, research team collaborators in Mexico made a cultural adaptation of the scales considering the variations of the Spanish spoken in Mexico.

To measure PD, the Kessler Psychological Distress Scale K10 was used (Kessler and Mroczek, 1994; Alonso et al., 2010; Mewton et al., 2016; Castro et al., 2019). This scale was designed by Kessler and Mroczek (1994) and it is composed of 10 items (i.e., “During the last 30 days, about how often did you feel depressed?”) and offers an overall score of PD. There are five response options (none of the time, a little of the time, some of the time, most of the time, and all of the time). The possible scores range between 10 and 50. Scores can be classified into four categories: “no psychological distress” (scores of 10–19), “slight psychological distress” (score of 20–24), “moderate psychological distress” (25–29), and “extreme psychological distress” (30–50). The scale has been shown to have adequate psychometric properties: [SBχ^2^ = 293.4076, df = 29, *p* < 0.001, CFI = 0.979, RMSEA = 0.049 (0.044, 0.055)]. Factor loadings ranged between 0.65 and 0.77. The scale offers good internal consistency, MacDonald’s omega coefficient of the scale was 0.91.

To measure PNCSR, the Reputation Enhancement Scale was used (RES; Carroll et al., 1999; Buelga et al., 2012; Del Moral et al., 2019; Jiménez et al., 2019). The social reputation scale was originally designed by Carroll et al. (1999) to obtain information regarding the non-conformist self-perception of adolescents. This scale consists of 15 items, each with four response options (never, rarely, many times, and always) and presents three dimensions that measure the adolescents’ self-perception of their social reputation: non-conformist self-perception, conformist self-perception, and self-perception of reputation. For the present study, the non-conformist self-perception dimension (items 2, 5, 6, 7, 9, 12, and 13) was used (“I would like others to think I am a rebellious child”). The CFA confirmed an adequate fit of the model to the data: [SBχ^2^ = 530.3886, df = 55, *p* < 0.001, CFI = 0.930, RMSEA = 0.048 (0.044, 0.052)]. Factor loadings ranged between 0.59 and 0.82. MacDonald’s omega coefficients of the scale and subscales were 0.93 (RES), 0.88 (non-conformist self-perception subscale), 0.75 (conformist self-perception subscale), and 0.75 (self-perception of reputation subscale).

To measure FC, the Parent-Adolescent Communication Scale (PACS) was used (Barnes and Olson, 1982; Jiménez et al., 2009, 2019; Cava, 2011). This instrument was developed by Barnes and Olson (1982) and consists of two sub-scales, one referring to children’s communication with the mother and the other to communication with the father. Both scales contain 20 items, which are grouped into two dimensions: open communication (items 1, 2, 3, 6, 7, 8, 9, 13, 14, 16, and 17; i.e., “My mother/father tries to understand my point of view”) and problematic communication, which includes items related to offensive communication (items 5, 12, 18, and 19; i.e., “My mother/father has a tendency to say things to me which would be better left unsaid”) and avoidable communication (items 4, 10, 11, 15, and 20; i.e., “When we are having a problem, I often give my mother/father the silent treatment”). Fit indices of the CFA were determined as follows: [SBχ^2^ = 1628.2179, df = 140, *p* < 0.001, CFI = 0.942, RMSEA = 0.053 (0.051, 0.056)]. Factor loadings ranged between 0.59 and 0.84. MacDonald’s omega coefficients of the scale and subscales were 0.95 (FC scale), 0.92 (open communication subscale), and 0.88 (problematic communication subscale).

To measure PUSNS, the problematic use of SNS in adolescence scale was used (Martínez-Ferrer et al., 2018a,b). This instrument was designed by Martínez-Ferrer et al. (2018b) to measure the problematic use of social networks using a scale of 13 items (i.e., “I need to be connected to my social networks continuously”), with response options from 1 (never) to 4 (always). The CFA confirmed an adequate fit of the model to the data: [SBχ^2^ = 20.8770, df = 2, *p* < 0.001, CFI = 0.990, RMSEA = 0.050 (0.032, 0.071)]. Factor loadings ranged between 0.67 and 0.80. MacDonald’s omega coefficient of the scale was 0.81.

To measure CPV, the Conflict Tactics Scale (CTS2) was used (Straus and Douglas, 2004; Gámez-Guadix et al., 2012; Del Moral et al., 2019; Suárez-Relinque et al., 2019). CTS2 is an instrument designed originally by Straus et al. (1996) to measure the extent to which partners engage in verbal and physical attacks on each other. In recent years, several authors have adapted the scale to analyze the violence exerted by adolescents toward his/her parents (see Gámez-Guadix et al., 2012; Suárez-Relinque et al., 2019). In the present study, we used the adaptation developed by Gámez-Guadix et al. (2012) to measure this type of violence in adolescents. The scale offers a global index of child-to-parent violence and scores in two dimensions (verbal aggression and physical assault). Items 1–3 reflect verbal aggression (i.e., “I insult or have insulted or sworn at my parents”) while items 4–6 reflect physical assault (i.e., “I slap, hit or have slapped or hit my parents”). Adolescents have to respond twice to each item (one for the mother and one for the father), taking into account the last year. The scale used by Gámez-Guadix et al. (2012) included a response scale with 7 options (0 = never to 6 = more than 20 times). In the present study, the instrument was adapted using a response scale composed by 5 points (0 = never to 4 = many times). The scale has been shown to have excellent psychometric properties: [SBχ^2^ = 33.8854, df = 12, *p* < 0.001, CFI = 0.965, RMSEA = 0.022 (0.014, 0.031)]. Factor loadings ranged between 0.64 and 0.80. MacDonald’s omega coefficients of the scales and subscales were: 0.88 (complete scale), 0.75 (verbal aggression subscale), and 0.82 (physical assault subscale) respectively.

### Data Analysis

Statistical analysis in the present study was carried out using SPSS software (version 20.0; IBM, Armonk, NY), except the Confirmatory Factor Analysis (CFA), which was conducted using EQS 6.1. First, to evidence the validity of the study scales in the Mexican adolescent population, a CFA was performed. McDonald’s omega coefficient was calculated to measure the internal consistency of the scales and subscales used in the study. Second, the scores of the boys and girls were analyzed to check for sex-based differences. For this purpose, an exploratory analysis was carried out using descriptive statistics (M and SD) and a means contrast (Student’s T) for the different study variables. In the latter case, Levene’s test for equality of variances was taken into account in the application of the contrast test. Also, to check the assumption of normality, the Kolmogorov-Smirnov test was used. Non-significant result was obtained from the test confirming the normal distribution of the data. Third, Pearson’s correlations between all the study variables were calculated. Finally, to estimate the relative weight of predictor variables, a multiple stepwise regression analysis was carried out for the total sample and for boys and girls separately.

## Results

As shown in Table 2, girls of the study obtained higher scores in CPV, VCPV, PD, and PUSNS while boys registered higher scores in PCPV and PNCSR. Also, girls showed higher levels than boys in most dimensions of FC (OCM, PCF, and PCM), while boys obtained higher scores in the case of OCF. Results of the *T*-test pointed statistically significant differences in CPV according to sex. Significant differences between boys and girls were also observed in VCPV, PUSNS, PNCSR, PD, PCM, OCF, and PCF, but no sex-based differences were observed in PCPV and OCM. On the other hand, considering the size of the effect, the significant differences obtained according to sex were relevant only in the case of VCPV (small effect), PUSNS (small effect), and PD (medium effect). The size of the effect showed no relevant differences between boys and girls in CPV, PNCSR, PCM, OCF, and PCF.

**Table 2:** Means, standard deviations, and differences (*T*-test) for the study variables according to sex.

	Sex	M	SD	Levene’s test		*T*-test	d
				F	Sig.	T	
CPV	Male	1.2081	0.31402	19.213	0.000	−6.784^***^	−0.070
	Female	1.2784	0.31670				
PCPV	Male	1.0635	0.25896	0.427	0.513	−0.488	--
	Female	1.0675	0.24631				
VCPV	Male	1.4771	0.56121	83.486	0.000	−10.688^***^	−0.225
	Female	1.7017	0.70004				
PUSNS	Male	1.7214	0.59867	64.010	0.000	−11.828^***^	−0.258
	Female	1.9790	0.71346				
PNCSR	Male	1.4869	0.50293	4.347	0.037	4.837^***^	0.079
	Female	1.4079	0.49184				
PD	Male	1.8649	0.75031	72.793	0.000	−18.797^***^	−0.512
	Female	2.3767	0.89052				
OCM	Male	3.5256	1.06441	3.169	0.075	−1.370	--
	Female	3.5738	1.08027				
PCM	Male	1.9629	0.74408	7.497	0.006	−7.407^***^	−0.183
	Female	2.1455	0.75629				
OCF	Male	3.2839	1.07846	0.104	0.747	5.273^***^	0.187
	Female	3.0972	1.07727				
PCF	Male	1.9062	0.77136	4.243	0.039	−4.887^***^	−0.126
	Female	2.0320	0.79453				

CPV, child-to-parent violence; PCPV, physical child-to-parent violence; VCPV, verbal child-to-parent violence; PUSNS, problematic use of social networking sites; PNCSR, perceived non-conformist social reputation; PD, psychological distress; OCM, open communication with the mother; PCM, problematic communication with the mother; OCF, open communication with the father; PCF, problematic communication with the father.

*** *p* < 0.001.

Pearson’s correlations between all the study variables were calculated (Table 3). Most of the correlations were statistically significant. In the case of CPV, the highest correlations were observed with both types of violence PCPV (*r* = 0.723) and VCPV (*r* = 0.888), with PNCSR (*r* = 0.388) and PD (*r* = 0.372), and the lowest with OCM (*r* = −0.165) and OCF (*r* = −0.198). The highest correlations in the table were observed between PCF and PCM (*r* = 0.655) and between OCF and OCM (*r* = 0.649). No correlation was detected between PCF and OCF.

**Table 3:** Correlations among CPV dimensions, PUSNS, PNCSR, PD, and dimensions of FC.

	CPV	PCPV	VCPV	PUSNS	PNCSR	PD	OCM	PCM	OCF	PCF
CPV	1	0.723^**^	0.888^**^	0.324^**^	0.388^**^	0.372^**^	−0.165^**^	0.323^**^	−0.198^**^	0.278^**^
PCPV		1	0.451^**^	0.144^**^	0.262^**^	0.160^**^	−0.125^**^	0.182^**^	−0.112^**^	0.165^**^
VCPV			1	0.365^**^	0.373^**^	0.442^**^	−0.157^**^	0.352^**^	−0.220^**^	0.315^**^
PUSNS				1	0.297^**^	0.374^**^	−0.115^**^	0.269^**^	−0.115^**^	0.176^**^
PNCSR					1	0.241^**^	−0.163^**^	0.277^**^	−0.167^**^	0.200^**^
PD						1	−0.133^**^	0.367^**^	−0.197^**^	0.259^**^
OCM							1	−0.048^**^	0.649^**^	0.083^**^
PCM								1	−0.038^*^	0.655^**^
OCF									1	−0.025
PCF										1

CPV, child-to-parent violence; PCPV, physical child-to-parent violence; VCPV, verbal child-to parent violence; PUSNS, problematic use of social networking sites; PNCSR, perceived non-conformist social reputation; PD, psychological distress; OCM, open communication with the mother; PCM, problematic communication with the mother; OCF, open communication with the father; PCF, problematic communication with the father.

** Correlation is significant at the 0.01 level (bilateral).

* Correlation is significant at the 0.05 level (bilateral).

To estimate the relative weight of predictor variables, a stepwise regression analysis was performed considering the total sample (Table 4). In the first step, the PUSNS variable was included. The model obtained was significant *F*(1, 3,729) = 438.525, *p* < 0.001. PUSNS (β = 0.324; *p* < 0.001) explained 10.5% of the variance in CPV (R^2^ = 0.105). In the second step, the PNCSR variable was included. PUSNS (β = 0.229; *p* < 0.001), together with PNCSR (β = 0.320; *p* < 0.001), contributed to the prediction of the model *F*(2, 3,728) = 461.540, *p* < 0.001, which explained 19.8% of the variance. Regarding the third step, the PD variable was included. In this case, PUSNS (β = 0.147; *p* < 0.001) and PNCSR (β = 0.284; *p* < 0.001), together with PD (β = 0.249; *p* < 0.001), contributed to the prediction of the model *F*(3, 3,727) = 415.538, *p* < 0.001, which explained 25.1% of the variance. Finally, in the fourth step, the dimensions of FC were included. In this last step it was observed that PUSNS (β = 0.129; *p* < 0.001), PNCSR (β = 0.242; *p* < 0.001), and PD (β = 0.190; *p* < 0.001), together with OCM (β = −0.047; *p* < 0.05), PCM (β = 0.074; *p* < 0.001), OCF (β = −0.070; *p* < 0.001), and PCF (β = 0.112; *p* < 0.001) contributed to the prediction of the model *F*(7, 3,723) = 209.746, *p* < 0.001, which explained 28.3% of the variance of CPV.

**Table 4:** Stepwise linear regression analysis (total sample).

Variables	B	Standard error	Beta	*p*	R^2^
Step 1					0.105
PUSNS	0.154	0.007	0.324	0.000^***^	
Step 2					0.198
PUSNS	0.109	0.007	0.229	0.000^***^	
PNCSR	0.203	0.010	0.320	0.000^***^	
Step 3					0.251
PUSNS	0.070	0.007	0.147	0.000^***^	
PNCSR	0.181	0.010	0.284	0.000^***^	
PD	0.092	0.006	0.249	0.000^***^	
Step 4					0.283
PUSNS	0.061	0.007	0.129	0.000^***^	
PNCSR	0.154	0.010	0.242	0.000^***^	
PD	0.070	0.006	0.190	0.000^***^	
OCM	−0.014	0.006	−0.047	0.013^*^	
PCM	0.031	0.008	0.074	0.000^***^	
OCF	−0.020	0.005	−0.070	0.000^***^	
PCF	0.045	0.008	0.112	0.000^***^	

CPV, child-to-parent violence; PUSNS, problematic use of social networking sites; PNCSR, perceived non-conformist social reputation; PD, psychological distress; OCM, open communication with the mother; PCM, problematic communication with the mother; OCF, open communication with the father; PCF, problematic communication with the father.

* *p* < 0.05

*** *p* < 0.001.

In order to explore the differences in the predictive weight of the variables according to sex, a multiple stepwise regression analysis was carried out separately for boys (Table 5) and girls (Table 6).

**Table 5:** Stepwise linear regression analysis (male subsample).

Variables	B	Standard error	Beta	*p*	R^2^
Step 1					0.065
PUSNS	0.134	0.011	0.255	0.000^***^	
Step 2					0.155
PUSNS	0.086	0.011	0.164	0.000^***^	
PNCSR	0.196	0.013	0.313	0.000^***^	
Step 3					0.194
PUSNS	0.059	0.011	0.112	0.000^***^	
PNCSR	0.174	0.013	0.279	0.000^***^	
PD	0.088	0.009	0.210	0.000^***^	
Step 4					0.221
PUSNS	0.051	0.011	0.097	0.000^***^	
PNCSR	0.149	0.013	0.238	0.000^***^	
PD	0.073	0.009	0.173	0.000^***^	
OCM	−0.011	0.008	−0.038	0.169	
PCM	0.032	0.012	0.077	0.009^**^	
OCF	−0.029	0.008	−0.098	0.000^***^	
PCF	0.029	0.012	0.072	0.013^*^	

CPV, child-to-parent violence; PUSNS, problematic use of social networking sites; PNCSR, perceived non-conformist social reputation; PD, psychological distress; OCM, open communication with the mother; PCM, problematic communication with the mother; OCF, open communication with the father; PCF, problematic communication with the father.

* *p* < 0.05

** *p* < 0.01

*** *p* < 0.001.

**Table 6:** Stepwise linear regression analysis (female subsample).

Variables	B	Standard error	Beta	*p*	R^2^
Step 1					0.135
PUSNS	0.163	0.010	0.368	0.000^***^	
Step 2					0.250
PUSNS	0.106	0.010	0.239	0.000^***^	
PNCSR	0.234	0.014	0.363	0.000^***^	
Step 3					0.300
PUSNS	0.074	0.010	0.166	0.000^***^	
PNCSR	0.199	0.014	0.309	0.000^***^	
PD	0.087	0.008	0.246	0.000^***^	
Step 4					0.344
PUSNS	0.065	0.010	0.147	0.000^***^	
PNCSR	0.169	0.014	0.263	0.000^***^	
PD	0.060	0.008	0.168	0.000^***^	
OCM	−0.017	0.008	−0.059	0.025^*^	
PCM	0.034	0.011	0.081	0.002^**^	
OCF	−0.006	0.008	−0.021	0.409	
PCF	0.064	0.010	0.161	0.000^***^	

CPV, child-to-parent violence; PUSNS, problematic use of social networking sites; PNCSR, perceived non-conformist social reputation; PD, psychological distress; OCM, open communication with the mother; PCM, problematic communication with the mother; OCF, open communication with the father; PCF, problematic communication with the father.

* *p* < 0.05

** *p* < 0.01

*** *p* < 0.001.

### Stepwise Regression (Boys)

In the first step, the PUSNS variable was included. The model obtained was significant *F*(1, 2011) = 139.552, *p* < 0.001. PUSNS (β = 0.255; *p* < 0.001) explained 6.5% of the variance in CPV (R^2^ = 0.065). In the second step, the PNCSR variable was included. PUSNS (β = 0.164; *p* < 0.001), together with PNCSR (β = 0.313; *p* < 0.001), contributed to the prediction of the model *F*(2, 2010) = 184.355, *p* < 0.001, which explained 15.5% of the variance. Regarding the third step, the PD variable was included. In this case, PUSNS (β = 0.112; *p* < 0.001) and PNCSR (β = 0.279; *p* < 0.001), together with PD (β = 0.210; *p* < 0.001), contributed to the prediction of the model *F*(3, 2009) = 161.486, *p* < 0.001, which explained 19.4% of the variance. Finally, in the fourth step, the dimensions of FC were included. In this last step it was observed that PUSNS (β = 0.097; *p* < 0.001), PNCSR (β = 0.238; *p* < 0.001), and PD (β = 0.173; *p* < 0.001), together with PCM (β = 0.077; *p* < 0.01), OCF (β = −0.098; *p* < 0.001), and PCF (β = 0.072; *p* < 0.05), contributed to the prediction of the model *F*(7, 2005) = 81.123, *p* < 0.001, which explained 22.1% of the variance of CPV.

### Stepwise Regression (Girls)

In the first step, the PUSNS variable was included. The model obtained was significant *F*(1, 1716) = 268.052, *p* < 0.001. PUSNS (β = 0.368; *p* < 0.001) explained 13.5% of the variance in CPV (R2 = 0.135). In the second step, the PNCSR variable was included. PUSNS (β = 0.239; *p* < 0.001), together with PNCSR (β = 0.363; *p* < 0.001), contributed to the prediction of the model *F*(2, 1715) = 286.170, *p* < 0.001, which explained 25% of the variance. Regarding the third step, the PD variable was included. In this case, PUSNS (β = 0.166; *p* < 0.001) and PNCSR (β = 0.309; *p* < 0.001), together with PD (β = 0.246; *p* < 0.001), contributed to the prediction of the model *F*(3, 1714) = 244.444, *p* < 0.001, which explained 30% of the variance. Finally, in the fourth step, the dimensions of FC were included. In this last step it was observed that PUSNS (β = 0.147; *p* < 0.001), PNCSR (β = 0.263; *p* < 0.001), and PD (β = 0.168; *p* < 0.001), together with OCM (β = −0.059; *p* < 0.05), PCM (β = 0.081; *p* < 0.01), and PCF (β = 0.161; *p* < 0.001), contributed to the prediction of the model *F*(7, 1710) = 128.351, *p* < 0.001, which explained 34.4% of the variance of CPV.

## Discussion

The main objective of this study was to identify the predictor variables of CPV in the adolescent’s individual and family environment, taking into consideration the possibility of there being differences based on sex. The results of our study confirmed the important role of PNCSR, PUSNS, PD, and FC as predictors of CPV and showed some important differences in the way these variables predict CPV depending on the sex of the adolescent.

Firstly, significant differences were observed in the scores of boys and girls in most of the study variables. In the case of CPV, girls showed a higher global index than boys. Also, girls of the study obtained higher scores in VCPV, however, no significant differences were detected between boys and girls in PCPV. These results point in the expected direction considering the sample used. In other terms, studies based on community samples have described a disparity of results that include higher levels of CPV in girls (Ibabe, 2015; Calvete and Veytia, 2018) and higher levels of verbal aggression in girls than in boys (Pagani et al., 2009; Calvete et al., 2013b; Jaureguizar et al., 2013; Calvete and Orue, 2016; Beckmann et al., 2017). Regarding this information, it is important to note that in community contexts, there is a lower presence of physical violence toward parents than of verbal violence. The difference in means of CPV in favor of girls obtained in studies that use community samples like the present study, is explained in many cases by the fact that girls obtain similar scores to boys in physical violence, but significantly higher scores on the verbal violence sub-scale (see Calvete et al., 2013b; Beckmann et al., 2017).

The results for the other variables revealed differences according to sex in the expected direction. The girls obtained higher scores in PD (Mewton et al., 2016; Van Droogenbroeck et al., 2018; Zhang et al., 2018) and PUSNS (Sarabia and Estévez, 2016; Martínez-Ferrer et al., 2018b; Aparicio et al., 2020), while higher scores were registered for boys in PNCSR (Buelga et al., 2012; Shin, 2017). In terms of FC, the girls showed higher levels in most dimensions: OCM, PCF, and PCM. In contrast, higher scores were observed in boys in the case of OCF. These findings are consistent with what is observed in recent studies (Parra and Oliva, 2002; Cava, 2003; Keijsers and Poulin, 2013). Finally, it is also important to indicate that despite sex-based differences were significant in most of the study variables, the size of these differences could be considered as relevant only in the case of VCPV, PUSNS, and PD.

Secondly, regarding the prediction analyses performed, it should be noted that all the variables included in the first model (considering the total sample) were significant predictors of CPV. As the study variables were included in the regression model, this increased the percentage of explained variance of the dependent variable. Even so, the variables that showed greater predictive importance in the set were PNCSR, PUSNS, and PD. When discussing our results, it is important to note that literature on the role of these individual variables in CPV is very scarce and the studies that have been conducted have focused mainly on the sphere of school violence (Buelga et al., 2012; Estévez et al., 2014).

In the case of PNCSR, the recent research by Del Moral et al. (2019) reported a positive correlation between PNCSR and CPV. Also, Terceño (2017) found that adolescents from families with high levels of CPV scored higher in PNCSR than those who came from families with medium and low level of CPV. In this sense, a positive correlation was also observed between both variables in the present study, together with their important predictive power. To explain these results, it is first important to consider the link established in prior research between PNCSR and violence in adolescence. Previous studies in the school context have reported that PNCSR represents a risk factor for adolescents’ participation in violent behaviors (Estévez et al., 2014; Buelga et al., 2015; Romero et al., 2019). It is important to remind that adolescents’ perception of their PNCSR is more favorable the more they perceive themselves as persons who defy rules and authority (Romero et al., 2019). In this sense, the main figures of authority normally confronted by adolescents in the school context are teachers, and adult parents or referents with whom they live in the family context. Taking into account the foregoing and the results of our study, PNCSR could be treated as a risk factor, not only in the school context but also in the case of CPV.

The second variable to show a greater capacity to predict CPV was PUSNS. Although a decrease in its predictive value was observed as the rest of the variables were included in the regression analysis, it proved to be one of the most relevant variables in the model. Again, little theoretical background information was found when interpreting our results. One of the few studies to present data on the relationship between PUSNS and CPV is the one by Martínez-Ferrer et al. (2018a). The aforementioned study showed that the higher the PUSNS scores, the higher the levels of CPV observed in adolescents. In this sense, our findings are consistent with those published in the aforementioned study, and PUSNS was observed to be positively correlated with adolescent violence toward parents, as well as being one of the most important predictors of the regression model. It is important to note that PUSNS has been routinely linked to violent behavior with peers. This relationship seems to be modulated, as suggested by Martínez-Ferrer et al. (2018a), by a positive attitude toward the transgression of social norms. In this sense, in the present study PNCSR and PUSNS accounted for around 20% of the variance in CPV, which would, to a certain extent, endorse the hypothesis proposed by the aforementioned authors for CPV; hence, the need to continue investigating the relationship between social reputation, the problematic use of social networks and the different forms of violence in adolescence.

The results obtained in the present study also showed how the model significantly increases its capacity to predict CPV when PD is included. In other words, according to the results obtained here, experiencing symptoms of anxiety and depression in adolescence increases the likelihood of assaulting parents or authority figures in the family. Previous literature has confirmed the relationship between PD and violence in adolescence, mainly in the field of peers. Although results vary, some researchers have reported higher levels of PD in adolescents who attack their peers compared to ordinary adolescents (Carlson and Corcoran, 2001; Sánchez-Sosa et al., 2010). As regards the study of the relationship between PD and CPV, very few studies have examined this aspect in literature. Nevertheless, in the research conducted by Lozano et al. (2013), a positive correlation was observed between both variables. Kennedy et al. (2010) found that adolescents who were violent toward their parents had experienced greater PD than those who were not. Also, results from the qualitative study carried out by Calvete et al. (2014a) pointed to emotional stress in children as a relevant predictor of CPV. Our results would be in line with those described in the abovementioned studies. According to our research, higher PD levels would coincide with higher CPV scores, and PD could be considered an important variable when predicting violence against parents in adolescence. Although our results are interesting and relevant, there is still little evidence in scientific literature regarding the role of PD in CPV to draw clear conclusions, and future research will need to study this relationship in greater depth.

The present study also analyzed the role of FC in CPV. Here, problematic communication significantly predicted the observed increases in CPV, especially in the case of the father, while the open communication predicted the decrease in CPV levels. Therefore, these results suggest that problematic communication, namely the form characterized by humiliating comments, threats, blame, insults, and screaming, is a risk factor for the development of CPV in adolescence, whereas open communication, characterized by spontaneity, listening and acceptance, is a protective factor. This result goes in the direction of what was obtained by Contreras and Cano-Lozano (2014a). Notwithstanding the foregoing, it is important to note that with the exception of the research developed by Contreras and Cano-Lozano (2014a), most of previous studies have analyzed the role of FC as an integral aspect of the study of parental socialization practices (Beckmann et al., 2017; García et al., 2018). In other words, there is a lack of information in scientific literature regarding the specific role played by the dimensions of FC (problematic and open communication) in the development of CPV in adolescence. We therefore believe that our results are interesting and make a relevant contribution to the study of CPV.

Finally, regarding sex-based differences, relevant information was obtained from the results of the multiple stepwise regression analysis carried out for boys and girls separately. First, we observed that the predictive capacity of the regression model was higher in girls than in boys. The analysis also confirmed the important role of individual variables (PNCSR, PUSNS, and PD) as predictors of CPV in boys and girls. PNCSR and PD showed similar values in both models and the main difference between girls and boys was observed regarding the predictive weight of PUSNS, significantly higher in girls than in boys. This last result goes in the direction of what it is shown in the study developed by Martínez-Ferrer et al. (2018a), and points to PUSNS as being a risk factor for CPV especially relevant for girls.

On the other hand, some important differences were detected in the results of boys and girls with respect to FC dimensions. Regarding open communication, OCF shows as a significant factor for predicting the decrease of CPV levels for boys, but not for girls. Conversely, OCM predicted the decrease of CPV for the girls of the study, but not for the boys. These results should be underlined as they provide relevant information for prevention strategies, namely that OCF and OCM should not be considered as protective factors without taking into account the sex of the adolescent.

In the case of problematic communication, PCM and PCF contributed significantly to predict CPV in boys and girls. However, while similar values were obtained in the case of PCM in both boys and girls (slightly higher in girls), the predictive weight of PCF was significantly higher in girls than in boys. In order to interpret this result, we should consider several aspects contrasted in the relevant literature. First, girls show higher levels of FC than boys and are more sensitive to family conflicts (Romero-Abrio et al., 2019). Second, the differential socialization of boys and girls in the family and its relationship with CPV must be taken into account (Cortina and Martin, 2020). For example, conflicts related to personal autonomy and independence are common during adolescence, especially in the case of girls who suffer more than boys from family restrictions that limit their freedom of conduct (Alonso-Stuyck and Aliaga, 2017). These kinds of conflicts tend to be solved in many cases unilaterally through parental imposition (López-Martínez et al., 2019). These considerations offer the beginnings of a possible explanation for why girls show higher levels than boys in PCF and PCM, and also for why girls are more affected in terms of CPV than boys through problematic communication with both parents (mainly with the father, who represents the prime authority figure). In other terms, the hypothesis could be that girls are more involved in family conflicts than boys due to the sex-based differences in socialization; girls are also more sensitive to family conflicts and show higher levels of FC than boys. Consequently, girls not only suffer more discomfort and frustration but also generate more arguments and engage in more violence (mainly verbal) toward parents than boys (López-Martínez et al., 2019; Cortina and Martin, 2020).

Summing up, the results of the present study confirm the role of PNCSR, PUSNS, PD, and FC as predictors of CPV and show some important differences in the way this set of variables predict CPV depending on the gender of the adolescent. First, PNCSR and PD showed similar values in boys and girls. Second, the main difference between boys and girls was observed in the predictive weight of PUSNS, which was higher in girls than in boys. Third, OCM appears as a preventive factor against CPV in the case of girls, while OCF does in boys. Fourth, although the two dimensions of problematic communication (PCM and PCF) could be considered as risk factors for boys and girls, the present research shows that both, but especially PCF, have a greater impact on girls.

These results have important implications for prevention: they reveal variables at both individual and family levels (the latter in the case of problematic communication) that can be risk factors for the development of CPV in adolescence. The results further point to the importance of open communication as a protective factor, to the importance of taking the sex of the adolescent into consideration, and lastly, to the role of communication with the mother, and with the father separately, when designing preventive strategies.

Finally, as pointed out earlier, there is no available information in recent research regarding the specific role played by problematic communication and open communication with the mother and the father in the development of CPV in adolescence. The findings and conclusions of our work clearly need further research.

One of the most relevant contributions of this study is the information provided in relation to variables that have been analyzed mainly in the sphere of violence between peers, but which have scarcely been studied in connection with CPV. This study provides information that reinforces the idea endorsed by some researchers (see Carrascosa et al., 2018), regarding the existence of a link between violence between peers and CPV. We verified that the individual and family dimensions with confirmed importance in violence between peers seem to play a similar role in CPV. We also consider that this idea of a general aggressor responding violently in different areas of his/her life due to the same variables is an exciting contribution that should be an important topic for future studies.

Nevertheless, this study had certain limitations that need to be highlighted. For example, according to our results, being a girl would imply a greater likelihood of engaging in violent behavior toward parents. This result is consistent with the findings reported in prior literature but should be interpreted with caution. Studies with large population samples such as ours have reported a greater presence of verbal violence (more common in girls), which may have an impact on the higher overall rate of CPV in the case of women. Therefore, the higher probability observed in girls could partly be explained by the type of sample chosen. Second, not only the overall CPV index but also the differences observed in the dependent variable (CPV) as a function of sex could have been explained with greater precision if the specific type of violence had been considered. Also, all the participants in the sample were selected in the age range corresponding to middle adolescence, as this is the stage in which most CPV cases are recorded. Nevertheless, we believe that the information provided here could be enriched through an analysis of the potential differences according to the specific stage of adolescence (early, middle, and late) in each individual. Lastly, it is worthwhile mentioning that this was a cross-sectional study in which causal relationships could not be established.

However, despite the abovementioned limitations, this study provides interesting and relevant information that should be considered in the field of prevention and for the development of future research.

## Data Availability Statement

The original contributions presented in the study are included in the article/supplementary material, further inquiries can be directed to the corresponding author.

## Ethics Statement

The studies involving human participants were reviewed and approved by Ethics Committee of the Pablo de Olavide University in Seville. Written informed consent to participate in this study was provided by the participants’ legal guardian/next of kin.

## Author Contributions

CS-R: conceptualization and writing—original draft preparation. CS-R, GM, and JC: methodology. CS-R and JC: software. CS-R and GM: formal analysis and funding acquisition. CS-R, GM, TJ, and JS: investigation. CS-R, GM, and TJ: writing—review and editing. GM, TJ, JC, and JS: supervision. All authors contributed to the article and approved the submitted version.

### Conflict of Interest

The authors declare that the research was conducted in the absence of any commercial or financial relationships that could be construed as a potential conflict of interest.
